# Alcohol-Induced Acute Liver Disease in Mice: A Comparison of the Preventive Effects of Fermented Milk from *Lactobacillus delbrueckii* Subsp. *bulgaricus* or *Lacticaseibacillus casei*

**DOI:** 10.3390/foods15071260

**Published:** 2026-04-07

**Authors:** Mingzhen Liu, Weimei Kong, Tao Zhang, Zhen Wu, Xiaoqun Zeng, Yuxing Guo, Daodong Pan

**Affiliations:** 1 Key Laboratory for Food Microbiology and Nutritional of Zhejiang Province, College of Food Science and Engineering, Ningbo University, Ningbo 315211, China; liumingzhen0706@163.com (M.L.); zhangtao@nbu.edu.cn (T.Z.); woodsen@163.com (Z.W.); zengxiaoqun@nbu.edu.cn (X.Z.); 2 Department of Food Science and Technology, School of Food Science and Pharmaceutical Engineering, Nanjing Normal University, Nanjing 210097, China; kongweimei2023@163.com

**Keywords:** *Lactobacillus*, alcohol-induced liver disease, fermented milk, protein expression, gut microbiome, gut–liver axis

## Abstract

Fermented milk is rich in probiotics, peptides, vitamins, and minerals, which are used as routine food supplements and are of great benefit for regulating human health. This study explored the mechanism of *Lactobacillus delbrueckii* ssp. *bulgaricus* CGMCC 21287 or *Lacticaseibacillus casei* CGMCC 15956 fermented milk for alleviating acute alcoholic liver injury. We found that fermented milk was associated with reduced activation of TLR4/NF-κB pathways, alleviating alcohol-induced liver inflammation. Meanwhile, the two probiotics regulated different intestinal microbial communities in mice. The LC group specifically increased the abundance of probiotics such as *Roseburia*, *unidentified_Lachnospiraceae*, and *Allobaculum*, and decreased the abundance of pathogenic bacteria such as *Enterococcus* and *Shigella*. The LB group increased the abundance of *Adlercreutzia* and *Ruminococcus*, thereby increasing butyric acid, acetic acid, and valeric acid levels and decreasing lipopolysaccharide (LPS) production. These results suggest that daily intake of fermented milk can attenuate alcohol-induced acute liver injury in mice via the gut–liver axis, though differences exist in the mechanisms of action and areas of emphasis.

## 1. Introduction

Environmental dangers and lifestyle factors, such as pollution and drug and alcohol addiction, put the liver at increased risk [1]. Alcohol abuse is one of the main factors that contributes to more than 200 diseases worldwide. Recent studies have shown that long-term use and over-consumption of alcohol causes excessive inflammation and oxidative stress, which eventually results in liver disease and even cancer [2]. Therefore, alcoholic liver injury is considered to be a serious worldwide health problem of great concern. It has been reported that a key factor speeding up alcohol liver disease (ALD) may be an imbalance of oxidative stress brought on by alcohol abuse, due to the toxic effects of oxidative stress on lipids, proteins, DNA, and RNA and its contribution to cellular dysfunction [3]. In recent years, the gut microbiome has received much attention due to its strong association with a range of diseases.

Increasingly, studies have revealed that alcohol intake disrupts the balance of the gut microbiota and leads to impaired intestinal integrity and barrier function [4]. The overgrowth of some pathogenic bacteria and the increase in intestinal permeability, significantly increases harmful metabolites (such as LPS) that enter the liver. Therefore, targeting the intestinal microbiota to prevent and control the progression of ALD may be an effective therapeutic approach, which in turn drives the development of new therapeutic approaches, including dietary modification, antibiotics, prebiotics, probiotics, and fecal microbiota transplantation [5]. Accumulating evidence has demonstrated the protective effects of specific lactic acid bacteria (LAB) strains against ALD. Supplementation with *Lactobacillus casei* Grx12 strain, derived from the gut microbiota of centenarians, has been found to protect rats from ALD by enhancing the expression of antioxidant enzymes and reducing pro-inflammatory cytokines [6]. Clinical evidence further supports these findings: a recent randomized controlled trial demonstrated that multi-strain probiotics (including *Lacticaseibacillus rhamnosus* LRa05 and *Bifidobacterium animalis* subsp. *lactis* BLa80) combined with conventional therapy significantly improved liver function markers (ALT, AST) and enriched beneficial genera such as *Bifidobacterium*, *Faecalibacterium*, and *Akkermansia* in ALD patients [7]. A systematic review and meta-analysis of 12 clinical trials further confirmed that probiotic supplementation significantly reduces ALT and AST levels while increasing the abundance of beneficial bacteria [8].

Fermented foods are widespread worldwide and can be considered as major vehicles for LAB, which may eventually affect host health by entering the gut microbiome. As an important part of traditional fermented food, LAB plays a decisive role in the quality and safety of fermented foods [9]. Generally speaking, the many enzymatic reactions that LAB will alter the composition of the foods in the process of food fermentation, producing bioactive compounds that can provide health-promoting characteristics that the same substrate would not display without fermentation [10]. Several studies have been conducted linking the consumption of fermented foods (mainly yogurt) with beneficial effects on human diseases [11]. Ingestion of *Bifidobacterium lactis Probio*-M8-fermented milk can reduce liver inflammation and oxidative stress, and regulate intestinal flora to alleviate alcoholic liver disease [12]. *Lactobacillus delbrueckii* ssp. *bulgaricus* is a well-known starter culture in yogurt production, while *Lacticaseibacillus casei* has demonstrated promising antioxidant capacity and probiotic properties in vitro. Despite the recognized potential of LAB in mitigating ALD, it remains unclear whether distinct probiotic strains mitigate this condition through differentiated mechanisms. This differentiation may stem from their unique functional characteristics, potentially involving the regulation of specific gut microbiota and associated host signaling pathways.

Therefore, the key objective of this study is to conduct a parallel comparative assessment of fermented milk products containing *Lactobacillus bulgaricus* subsp. *bulgaricus* CGMCC 21287 and *Lacticaseibacillus casei* CGMCC 15956 in an acute ALD mouse model, with a particular focus on elucidating their differential mechanisms of action via the gut–liver axis. By analyzing gut microbiota composition, intestinal tight junction protein expression, and hepatic oxidative stress and inflammation-related signaling pathways, this study aims to reveal the strain-dependent protective mechanisms of LAB-fermented milk against acute ALD. These findings may provide theoretical support for the targeted application of probiotic fermented foods in preventing alcoholic liver injury.

## 2. Materials and Methods

### 2.1. Functional Fermented Milk Preparation

*L. bulgaricus* and *L. casei* was obtained from traditional Chinese Xinjiang fermented cheese and stored in the China General Microorganism Culture Collection Center (CGMCC, Beijing, China) under ID number 21287 and 15956. *L. bulgaricus* CGMCC 21287 or *L. casei* CGMCC 15956 is mixed with sterilized skim milk and fermented at 42 °C for 4.5 h with an initial concentration of 5% (*v*/*v*). The pH of the fermented milk dropped to 4.48, the acidity value was above 80 °T, and the number of viable cells in the yogurt reached above 10^8^ CFU/mL. Moreover, the nutrient content of the milk medium (UHT Skim Milk, Devondale, Melbourne, Australia) used.

### 2.2. Animals and Treatments

The experimental protocol was approved by the Animal Experiment Center of Nanjing Normal University (SYXK (Su) 2015-0028) and followed relevant ethical regulations in this study. Sixty C57BL/6 male mice, which were 6-week-old and weighed 18–22 g, were bought. Mice were kept under a 12 ± 12 h light-dark cycle at an ambient room temperature of 22 ± 1 °C and relative humidity of 50 to 60%. Sixty mice were randomly assigned to five groups for the establishment of acute ALD caused by alcohol: 0.9% NaCl was given to healthy mice at a dose of 15 mL/kg per day (NG); acute ALD mouse model mice received the same dose of 0.9% NaCl (AG); *Lactobacillus bulgaricus* CGMCC 21287 fermented milk (LB), *L. casei* CGMCC 15956 fermented milk (LC) and glutathione (PC) was given to mice at a dose of 15 mL/kg per day. After 14 days of continuous administration of fermented milk or control treatments, all mice except those in the NG group received 56% alcohol (12 mL/kg) via gavage once daily for three consecutive days. During this period, serum alanine aminotransferase (ALT) and aspartate aminotransferase (AST) levels and liver HE staining results were used to determine whether the model was well established. Finally, all mice were fasted for 12 h and then sacrificed. Serum was collected from whole blood by centrifugation. The liver and intestinal samples were also collected from each group, and its weight relative to the final body weight was calculated using the following formula: Liver Index (mg/g) = Liver Weight (mg)/Body Weight (g). The fecal contents were collected in sterile tubes and all samples were stored at −80 °C until analysis, except for the liver used for histopathology which was fixed with 4% paraformaldehyde solution.

### 2.3. Measurement of Biochemical Indices

According to related kits, the activities of ALT, AST, lipopolysaccharide (LPS), liver triglyceride (TG), glutathione peroxidase (GPx), superoxide dismutase (SOD), malondialdehyde (MDA), interleukin-6 (IL-6), and tumor necrosis factor α (TNF-α) were determined (Jiancheng Bioengineering Institute, Nanjing, China). Additionally, the left hepatic lobe was embedded in paraffin after being fixed with 4% paraformaldehyde for more than 24 h. These were then cut into 5 m pieces and stained with hematoxylin and eosin (H&E). The liver slices were then observed using a microscope (Nikon-E100, NIKON, Japan). The liver injury scoring system comprised steatosis (0–3), lobular inflammation (0–2), and hepatocellular ballooning (0–2) [13].

### 2.4. Western Blotting Analysis

The BCA kit was used to determine the protein concentration of the samples after liver and intestinal cell lysates were obtained (Thermo, Rockford, IL, USA). The proteins were analyzed by SDS-PAGE using 5% concentrated gel and 15% separation gel before transferring them onto the NC membrane. The membranes were then incubated for an hour at 37 °C in a blocking solution that contained 5% skim milk powder. Then, specified primary antibodies (Occludin, Claudin, ZO-1, TLR4, p65, CYP2E1) were incubated with the membrane and left for an overnight period at 4 °C. Subsequently, the secondary antibody horseradish peroxidase (1:1000) was incubated with the membrane at room temperature for 1 h. The enhanced chemiluminescence (ECL) kit (Millipore, Darmstadt, Germany) luminescent solution was kept in the dark room on the front of the membrane for 5 min, and it was put into the imaging system (Tanon-5200, Tanon Science & Technology Co., Ltd., Shanghai, China) to detect protein bands and convert the chemiluminescence signal into a digital image for determining density.

### 2.5. 16s rRNA Gene Sequence Analysis

The total DNA was extracted from the cecum of mice, and the bacterial 16S rDNA hypervariable region V3-V4 was amplified by polymerase chain reaction (PCR). The PCR purification and sequencing was performed on the Illumina MiSeq platform by BionovoGene (Bio Tech Co., Ltd., Suzhou, China). Sequences with more than 97% similarity were assigned to the same operational taxonomic unit (OTU) by Vsearch (v2.3.4). Screened representative sequences of each OTU for further annotation. Samples were analyzed for α-diversity and β-diversity using QIIME software (version 1.7.0), and the community composition of each sample was counted.

### 2.6. Short-Chain Fatty Acids (SCFAs) Composition Analysis

SCFAs content in fecal samples was determined using the method described by [14]. Briefly, SCFAs were extracted and measured from the fecal contents of each animal by GC/MS. Chromatographic separation was performed on the HP-5 capillary column (30 m × 0.25 mm × 0.25 μm, Agilent Technologies, Santa Clara, CA, USA) and data processing was performed on the MSD ChemStation (E.02.00.493, Agilent Technologies, USA) by comparing the peak areas of the corresponding standards with the SCFAs of all samples. The Spearman correlation coefficients were used to analyze the relationships between gut microbial abundance (genus level) and SCFAs levels.

### 2.7. Statistical Analysis

GraphPad Prism (v. 11; GraphPad Software Inc., La Jolla, CA, USA) was used to analyze all data, which are expressed as means ± SD. Normality was evaluated using the Shapiro–Wilk test, and homogeneity of variances was examined using Bartlett’s test when normality assumptions were met or the Brown–Forsythe test when data deviated from normality. When both assumptions were satisfied, one-way ANOVA followed by Tukey’s HSD post hoc test was performed. R software (version 4.1.2) was used to report the results of the bioinformatics data analysis. Significance was set at *p* < 0.05.

## 3. Results

### 3.1. Fermented Milk Could Relieve Intestinal Damage Caused by Alcohol in Mice

**Figure 1:**
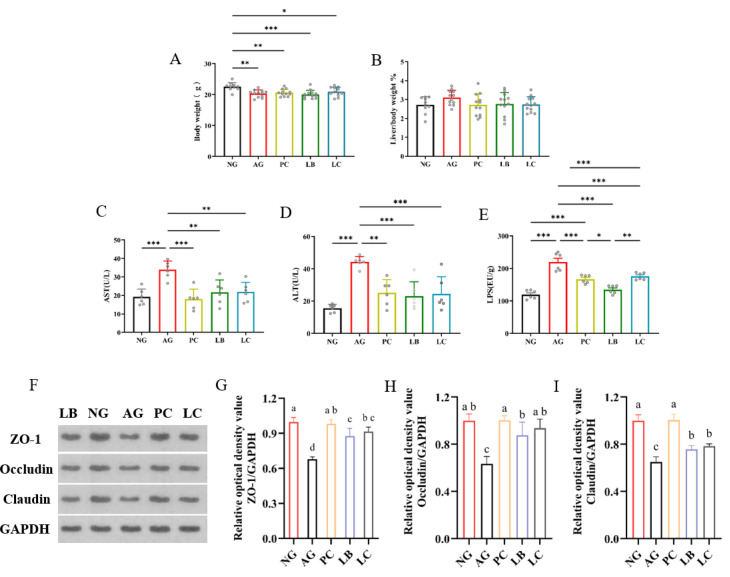
Effects of fermented milk treatment on (**A**) body weight; (**B**) liver/body weight; (**C**) serum AST levels; (**D**) ALT levels; (**E**) LPS levels (*n* = 6); (**F**–**I**) the protein expression of ZO-1, occludin, and claudin-1 in the ileum (*n* = 3). NG, normal group; AG, alcohol group; PC, glutathione group; LB, *L. bulgaricus* CGMCC 21287 fermented milk group; LC, and *L. casei* CGMCC 15956 fermented milk group. * *p* < 0.05, ** *p* < 0.01, *** *p* < 0.001; letters a–d indicate significant differences among groups (*p* < 0.05).

Body weight and liver/body ratio serve as sensitive indicators of mouse health status. As depicted in Figure 1A, mice in the alcohol gavage group exhibited significantly lower body weights than the NG group, indicating that repeated alcohol gavage over three consecutive days induced varying degrees of wasting or metabolic stress. Mice in the PC and LC group showed slightly higher body weights than the AG group (without a significant difference), suggesting a potential protective effect against alcohol-induced weight loss. Concurrently, the liver/body ratio in the AG group was significantly elevated compared to the NG group, reflecting the progression of acute ALD. Notably, the liver/body ratio in all treatment groups (PC, LB, and LC) was lower than that of the AG group, indicating a stronger mitigating effect on alcohol-induced hepatomegaly.

To evaluate liver damage, serum ALT and AST values are frequently used. In this study, the ALT and AST of the AG group were significantly higher than the NG group (Figure 1C,D), implying the successful establishment of the acute ALD model. However, ALT and AST levels were significantly reduced in the LB, LC and PC groups compared with the AG group (*p* < 0.05). In addition, the administration of LB and LC dramatically reduced alcohol-induced serum LPS levels (Figure 1E; *p* < 0.05).

Increased levels of LPS lead to impaired gut integrity and barrier function, triggering an inflammatory response. As shown in Figure 1F–I, the expression levels of ZO-1, occludin and claudin were significantly reduced after alcohol intake, indicating a disruption of intestinal barrier function. However, the expression levels of ZO-1, occludin and claudin were significantly increased in the treatment group compared to the AG group by gavage drug (*p* < 0.05). In particular, the PC group outperformed the LB and LC intervention groups and achieved an effect consistent with that of the NG group. Our results indicated that alcohol destroyed the integrity of the intestinal barrier. However, the intake of fermented milk can repair intestinal damage by increasing the expression level of intestinal tight junction proteins.

### 3.2. Fermented Milk Could Prevent Alcohol-Induced Liver Inflammation and Oxidative Stress Damage in Mice

**Figure 2:**
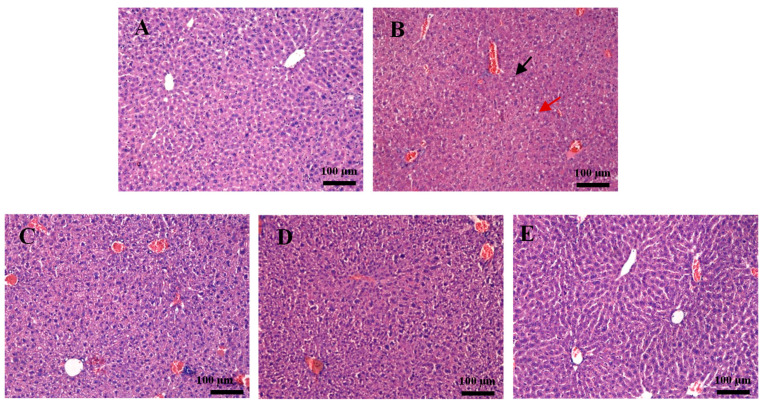
Liver histopathological changes in five different groups. (**A**) NG group; (**B**) AG group; (**C**) PC group; (**D**) LB group; (**E**) LC group.

Liver damage was usually indicated using HE staining and biochemical index levels. The results of liver pathological sections are shown in Figure 2. The hepatic lobules of the mice had a complete and distinct structure and the morphology of the cells was normal, with clear nucleoli and no obvious inflammatory infiltration in the NG group. However, the cells in the liver of the mice were severely swollen and deformed in the AG group; there were numerous inflammatory cells present between the liver sinusoids, and lipid droplets were observed in the tissue space. The PC, LB, and LC groups dramatically decreased the number of lipid droplets and inflammatory cell infiltration compared to the AG group.

**Figure 3:**
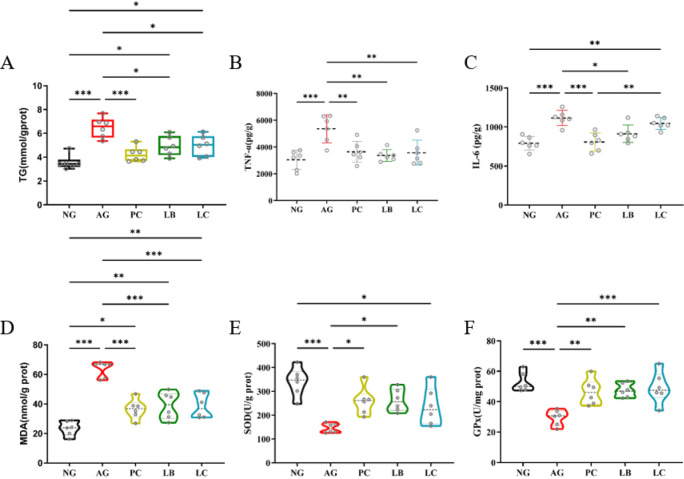
(**A**) Liver TG level; (**B**,**C**) liver TNF-α and IL-6 level; (**D**–**F**) liver MDA, SOD and GPX activity (*n* = 6). NG, normal group; AG, alcohol group; PC, glutathione group; LB, *L. bulgaricus* CGMCC 21287 fermented milk group; LC, and *L. casei* CGMCC 15956 fermented milk group. * *p* < 0.05, ** *p* < 0.01, *** *p* < 0.001.

The liver biochemical indicators showed that the contents of fat and inflammation were significantly increased in liver cells. For example, the levels of TG, IL-6, and TNF-α were higher than the NG group (Figure 3A–C). In addition, alcohol exposure also changed the level of oxidation stress in the hepatic of the mice, in which the level of lipid peroxide MDA increased significantly, while the level of SOD and GPX decreased (Figure 3D–F). In contrast, liver inflammation and oxidative stress induced by alcohol intake were alleviated in the LB and LC groups, except for the level of IL-6 in the LC group, which was not significantly different from that in the AG group. Similarly, the intervention effect of the PC group was similar to that of the LB group. This is consistent with the results of HE staining.

### 3.3. Fermented Milk Alleviates Alcohol-Induced Liver Inflammation in Mice by the NF-κB Signaling Pathway

**Figure 4:**
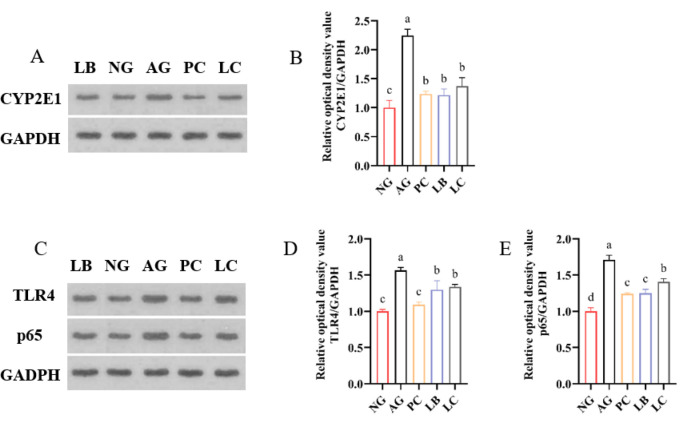
Effect of fermented milk treatment on the protein expression levels of (**A**,**B**) CYP2E1; (**C**–**E**) TLR4/NF-κB signaling pathway-related genes. NG, normal group; AG, alcohol group; PC, glutathione group; LB, *L. bulgaricus* CGMCC 21287 fermented milk group; LC, and *L. casei* CGMCC 15956 fermented milk group. The letters a–d indicate significant differences among groups (*p* < 0.05).

Ethanol intake can induce the overexpression of CYP2E1 protein, thereby producing a large amount of ROS and causing inflammation or oxidative imbalance in the body. As expected, alcohol intake increased CYP2E1 protein expression, and fermented milk and GSH intervention can reduce CYP2E1 protein expression (Figure 4A,B). Since ROS and gut-derived LPS can activate the NF-κB pathway in the liver to release inflammatory factors, so the level of TLR4 and NF-κB p65 protein expression was detected. According to the results, alcohol-intervention mice had considerably higher levels of NF-κB p65 and TLR4 protein expression in their livers than in the NG group, fermented milk and GSH treatment could attenuate this result. Direct statistical comparison revealed that the LC group exhibited significantly higher p65 expression than the LB group (*p* < 0.001), indicating that the LB treatment has superior potential in downregulating p65 compared to LC (Figure 4C–E).

### 3.4. Fermented Milk Could Modulate Gut Microbiota in Mice with Acute ALD

**Figure 5:**
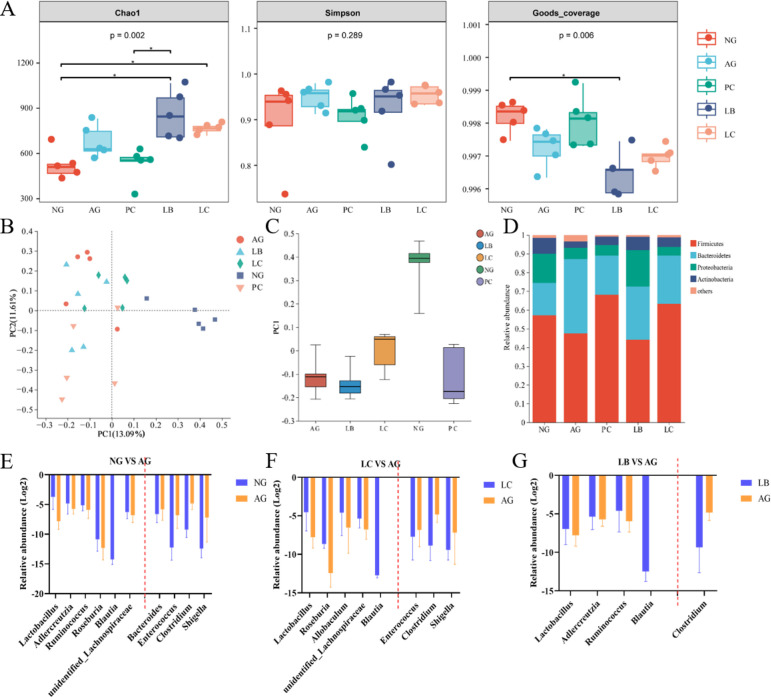
Effect of fermented milk treatment on gut microbiota. (**A**) Alpha diversity of five groups; (**B**) principal coordinate analysis (PCoA) plots; (**C**) box line plot of sample distances between the five groups; (**D**) gut microbiota composition at the phylum level; (**E**) differences in microorganisms between NG and AG groups at the genus level; (**F**) differences in microorganisms between LC and AG groups at the genus level; (**G**) differences in microorganisms between LB and AG groups at the genus level (*n* = 5). No *Blautia* was detected in the AG group (**E**–**G**). NG, normal group; AG, alcohol group; PC, glutathione group; LB, *L. bulgaricus* CGMCC 21287 fermented milk group; LC, and *L. casei* CGMCC 15956 fermented milk group.

The bacterial composition in fecal samples collected from each experimental group was characterized to determine the effects of fermented milk on the gut microbiota. Alpha diversity analysis revealed that the Chao1 index, which estimates microbial richness (the number of distinct taxa), was significantly increased in the LB and LC groups compared with the AG group (*p* < 0.05). In contrast, the Simpson index, which reflects species evenness (the uniformity of relative abundances among taxa), showed no significant difference among groups (*p* > 0.05). These results suggest that fermented milk intervention promoted the expansion of specific beneficial microbial taxa without substantially altering the overall evenness of the gut microbial community. In addition, the goods coverage values of all samples were higher than 0.994, indicating that the coverage rate of the samples in this sequencing was high (Figure 5A). The Bray–Curtis analysis showed that a higher degree of similarity between NG and LC groups compared to the NG vs. LB groups (Figure 5B,C).

Compared with the NG group, alcohol administration resulted in the increase in Bacteroidetes and the decrease in Firmicutes, Proteobacteria, and Actinobacteria at the phylum level (Figure 5D). LEfSe analysis was also performed to identify differentially abundant taxa across groups (Figure S2). Moreover, alcohol administration resulted in the decrease in *Lactobacillus*, *Adlercreutzia*, *Ruminococcus*, *Roseburia*, *Blautia*, and *unidentified_Lachnospiraceae* and the increase in *Enterococcus*, *Bacteroides*, *Clostridium*, and *Shigella* at the genus level (Figure 5E). However, the LC group can make the intestinal flora of the mice fed the alcohol group close to the normal group, and the effect was better than that of the LB and PC groups. Compared with the AG group, the LC group increased the relative abundance of *Lactobacillus*, *Roseburia*, *Blautia*, *Allobaculum*, and *unidentified_Lachnospiraceae* and decreased the relative abundance of *Enterococcus*, *Clostridium*, and *Shigella* (Figure 5F). Conversely, the LB group only increased the levels of *Lactobacillus*, *Adlercreutzia*, *Ruminococcus*, and *Blautia* and decreased the levels of *Clostridium* (Figure 5G).

### 3.5. Fermented Milk Could Increase Butyric Acid, Acetic Acid and Valeric Acid Levels in the Feces of Acute ALD Mice

**Figure 6:**
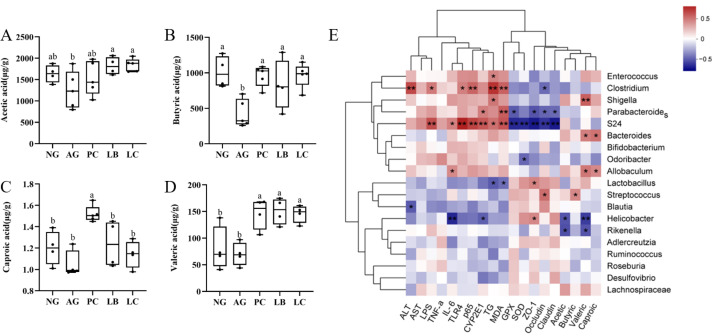
Effect of fermented milk treatment on the levels of short-chain fatty acids (SCFAs) in intestinal contents. (**A**) Acetic acid; (**B**) butyric acid; (**C**) caproic acid; and (**D**) valeric acid (*n* = 5); heatmap shows the Spearman correlations between ASVs at genus level and biochemical indicators (**E**). Significance was evaluated using the 2-tailed, unpaired Student *t*-test, * *p *< 0.05, ** *p *< 0.01. The letters a, b indicate significant differences among groups (*p* < 0.05). NG, normal group; AG, alcohol group; PC, glutathione group; LB, *L. bulgaricus* CGMCC 21287 fermented milk group; LC, and *L. casei* CGMCC 15956 fermented milk group.

SCFAs have been reported to exert beneficial effects on human health. As shown in Figure 6, comparing the AG group to the NG group, butyric acid levels were lower in the AG group. Furthermore, butyric acid, acetic acid and valeric acid levels were notably increased in the feces collected from the groups administered LB and LC groups. However, there was a substantial difference in butyric acid, caproic acid and valeric acid levels between the PC and AG groups. Spearman correlation analysis was performed to explore associations between gut microbial genera, SCFA levels, and biochemical parameters (Figure 6E). Several significant correlations were observed. Specifically, the abundance of *Enterococcus*, *Parabacteroides*, *Clostridium*, and *Shigella* was positively correlated with serum ALT, AST, LPS, and hepatic inflammatory cytokines (TNF-α, IL-6), and negatively correlated with antioxidant enzyme activities (SOD, GPx) and intestinal tight junction protein expression (ZO-1, occludin, claudin-1). Conversely, SCFAs levels (acetic acid, butyric acid, valeric acid) were positively correlated with the abundance of *Bacteroides*, *Lactobacillus*, *Streptococcus*, and *Allobaculum*, and negatively correlated with *Helicobacter* and *Rikenella.*

## 4. Discussion

This investigation systematically compared the protective effects and underlying mechanisms of *Lactobacillus delbrueckii* subsp. *bulgaricus* CGMCC 21287 and *Lacticaseibacillus casei* CGMCC 15956 fermented milk in a mouse model of acute ALD, both strains originating from traditional Xinjiang fermented cheese. This study demonstrates that although both LB and LC fermented milk effectively alleviate acute alcohol-induced liver injury, they exert differentially regulated modulation on hepatic inflammatory and antioxidant signaling pathways through remodeling distinct intestinal microbial communities. There are various approaches for achieving the necessary level of ALD. However, there is currently no approach that is widely used. In this study, a model of acute ALD was established by continuous gavage of 56% alcohol (12 mL/kg) for three days, following the method of Liang et al. with appropriate modifications [15]. Large amounts of alcohol over short periods of time can increase the permeability of liver cell membranes and cause hepatic enzyme leakage into the blood [16]. Therefore, significantly elevated serum levels of ALT and AST are the most commonly used indicators to assess the success of alcoholic liver injury. Our findings demonstrate the viability of the established model of acute ALD. The results of H&E staining also demonstrated alcohol-induced liver histopathological damage, including an increase in lipid droplet quantity and inflammatory cell infiltration.

Upon heavy alcohol consumption, a small portion is absorbed through the gastrointestinal tract, while the majority is metabolized in the liver via alcohol dehydrogenase and the microsomal ethanol-oxidizing system, with CYP2E1 playing a central role [17]. Excessive ethanol metabolism upregulates CYP2E1 expression, leading to the overproduction of reactive oxygen species (ROS) and the subsequent depletion of antioxidant defenses [18,19]. In our study, alcohol administration increased hepatic CYP2E1 expression and MDA levels while reducing antioxidant enzyme activities (SOD and GPx). Simultaneously, the acetaldehyde and ROS generated during this process also disrupt intestinal epithelial integrity, thereby facilitating the translocation of microbiota-derived products (particularly LPS)—from the gut to the liver [20]. The intestinal barrier is maintained by tight junction proteins such as ZO-1, occludin, and claudin-1, which regulate paracellular permeability [21]. In the present study, alcohol exposure significantly reduced the expression of these tight junction proteins and elevated serum LPS levels, consistent with previous reports demonstrating that ethanol compromises intestinal barrier function [22]. It is worth noting that following consumption of fermented milk containing *L. bulgaricus* or *L. casei*, the expression of tight junction proteins and oxidative stress levels were effectively restored, and serum endotoxin levels were reduced. This suggests that both strains can prevent alcohol-induced impairment of intestinal barrier function and hepatic oxidative stress. These findings align with earlier studies showing that specific lactic acid bacteria can enhance intestinal integrity by modulating tight junction protein expression [23,24]. Additionally, the protective effects are closely linked to the antioxidant properties of bioactive components produced during milk fermentation by lactic acid bacteria, such as exopolysaccharides (EPS) and peptides [25,26].

Concurrently, the translocation of gut-derived LPS to the liver activates Kupffer cells via TLR4, triggering a signaling cascade that leads to the nuclear translocation of NF-κB and the subsequent production of pro-inflammatory cytokines, including TNF-α and IL-6 [27]. Importantly, CYP2E1-derived ROS can directly activate NF-κB independent of TLR4 signaling, and can also sensitize hepatocytes to LPS-induced inflammation by amplifying TLR4-mediated signaling cascades [28]. This interplay between oxidative stress and LPS signaling establishes a synergistic feed-forward loop that exacerbates hepatic inflammation [29]. In this study, the effects of two fermented milk interventions on the activation of the TLR4/NF-κB pathway and on TNF-α and IL-6 levels in the liver were evaluated. Notably, although both LB and LC treatments reduced hepatic p65 expression compared with the AG group, the LC group exhibited significantly higher p65 levels than the LB group, which correlated with a lack of significant reduction in IL-6 levels in the LC group. There might be several reasons for this phenomenon. First, pro-inflammatory cytokines exhibit differential sensitivity to NF-κB inhibition. TNF-α is tightly regulated by NF-κB, whereas IL-6 can be sustained through NF-κB-independent mechanisms or requires more profound suppression of NF-κB for effective downregulation [30]. This is consistent with our observation. Second, LB and LC likely differ in their potency to suppress the TLR4/NF-κB signaling axis. LB intervention may more effectively inhibit upstream activators of NF-κB, such as TLR4 expression or MyD88 recruitment, resulting in stronger suppression of p65 nuclear translocation and subsequent IL-6 transcription [31]. In contrast, LC may only partially inhibit NF-κB activation, leaving residual signaling activity sufficient to sustain IL-6 expression. Third, the regulation of IL-6 expression is complex. Beyond the pathological IL-6 extensively secreted by hepatic Kupffer cells upon activation of the LPS/TLR4/NF-κB pathway, intestinal dendritic cells and lamina propria lymphocytes can secrete modest amounts of protective IL-6 upon stimulation through probiotics and their metabolites in the fermented milk [32]. Last, differences in gut microbiota regulation between the two groups may also account for this inconsistency. The different microbial communities enriched in the two types of fermented milk may produce distinct types of immunomodulatory metabolites, thereby leading to differences in NF-κB inhibition and downstream cytokine expression [33].

Moreover, an increasing amount of research has demonstrated a close relationship between the gut and liver, and gut microbes may also be involved in the development of metabolic diseases, referred to as the “gut–liver axis” [34]. Previous studies have demonstrated that the gut microbiota can influence liver pathology through mechanisms such as regulation of intestinal barrier integrity, bacterial translocation, and modulation of hepatic inflammatory responses [35,36]. In this study, alcohol exposure increases the level of *Enterococcus*, *Bacteroides, Clostridium* and *Shigella* in the intestine. The early liver cancer group had a lot of *Clostridiales* in their intestinal tract and *Shigella* has been shown to infiltrate and destroy M cells, as well as spread to neighboring enterocytes [37]. Furthermore, it was discovered that the primary cause of alcoholic liver damage was the exotoxin cytolysin released by *Enterococcus* [38]. Meanwhile, *Bacteroidetes* contains a large number of Gram-negative bacteria that can produce intestinal endotoxin LPS [39]. When LPS enters the liver through the damaged intestinal epithelial barrier and binds to the receptor TLR4, activating the NF-κB pathway and causing liver inflammation [40]. A large body of research has shown that dietary interventions, probiotics, or fermented products can simultaneously modulate the gut microbiota and ameliorate liver inflammation [41,42], which aligns with our observations. Notably, the observation that fermented milk increased Chao1 richness but did not affect Simpson evenness is noteworthy. This pattern aligns with previous probiotic intervention studies, where specific strains often induce targeted enrichment of certain beneficial genera rather than a global restructuring of the gut microbial ecosystem [43]. Consistent with this pattern, the taxonomic composition analysis revealed that the LB group mainly lowered *Clostridium* spp. and elevated *Adlercreutzia* spp. to alleviate inflammation, while the LC group mainly acted by lowering anti-inflammatory-related genera (*Enterococcus*, *Clostridium* and *Shigella*). *Adlercreutzia* is also a potential genus with anti-inflammatory effects [44].

Furthermore, fermented milk enters the large intestine where it is fermented, broken down and metabolized into absorbable metabolites that are beneficial to the body, such as SCFAs, which not only provide energy to the gut microbiota but also change the composition and physiological function of the microbiota [45]. In our study, genera with the ability to produce SCFAs such as *Lactobacillus*, *Blautia*, Roseburia, *unidentified_Lachnospiraceae,* and *Ruminococcus* were increased as a result of the treatment with fermented milk. As described, *Ruminococcus* can improve the integrity of the epithelial barrier and suppress inflammation through the production of butyric acid [46]. Butyrate, one of the most important SCFAs, exerts pleiotropic effects on host physiology. Mechanistically, butyrate functions as a histone deacetylase (HDAC) inhibitor, thereby promoting the differentiation of regulatory T cells (Tregs) in the gut, which is critical for maintaining immune homeostasis and suppressing excessive inflammatory responses [47]. Additionally, butyrate enhances intestinal barrier function by upregulating tight junction proteins such as claudin-1 and occludin, thereby reducing intestinal permeability and preventing barrier disruption [48]. Beyond its local effects in the gut, butyrate also systemically influences host metabolism; it has been shown to suppress hepatic lipogenesis by inhibiting the expression of key lipogenic enzymes, thereby ameliorating hepatic steatosis and improving metabolic profiles [49]. These multifaceted mechanisms underscore the beneficial role of butyrate-producing bacteria in host health. Treatment of mice with butyrate has been shown to prevent intestinal barrier damage, aligning with these mechanistic insights. It has been reported that *Allobaculum* was reported to be an active producer of butyrate [50]. *Blautia* spp. have been reported to affect host health with microbial products such as long-chain triglycerides and SCFAs, as well as participating in bile acid metabolism and promoting colonization resistance to intestinal pathogens [51]. This finding is in general agreement with the results of the SCFAs assay and correlation analysis. The levels of acetic acid and butyric acid in the fermented milk group matched those of the control group. This may be due to the fact that skimmed milk is fermented by *L. bulgaricus* 21287 or *L. casei* 15956 to produce small active ingredients that are more readily available to gut microbes [52]. It is well known that *Lactobacillus delbrueckii* ssp. *bulgaricus* is one of the widely applicable LAB in fermented milk products. Previous studies have found that *L. bulgaricus* shows protease or peptidase activity, suggesting that it may be better at hydrolyzing proteins into different oligopeptides [53]. Additionally, LAB can also produce a variety of vitamins and exopolysaccharide (EPS) in the process of fermenting milk, which has a good effect on intestinal flora and SCFAs [54]. Gao et al. found that fermentation would change the lipid profile and fatty acid composition of milk [55]. This may contribute to understanding the ability of fermented milk in reducing the risk of chronic diseases. Future studies will require larger sample sizes using fecal microbiota transplantation, targeted SCFA supplementation, and chronic or long-term alcohol feeding models to establish causality and validate the sustained hepatoprotective effects of fermented milk.

## 5. Conclusions

The functional fermented milk prepared by *L. bulgaricus* 21287 or *L. casei* 15956 showed better effects in the prevention of acute ALD in mice, including oxidative stress and inflammation in the liver, intestinal epithelial permeability, and gut microbiota. Additionally, daily intake of fermented milk could regulate alcohol-induced gut microbiota disturbance by increasing the relative abundance of *Lactobacillus*, *Blautia*, and/or *Roseburia*, *unidentified_Lachnospiraceae*, and *Ruminococcus* and reducing levels of *Clostridium* and/or *Enterococcus* and *Shigella*. This study not only confirmed the liver-protective potential of functional fermented milk but, more importantly, suggested the involvement of distinct mechanisms associated with different probiotic strains. This provides crucial theoretical foundations and strain resources for the future development of precision-targeted probiotic therapies or functional foods.

## Data Availability

The original contributions presented in this study are included in the article/Supplementary Materials. Further inquiries can be directed to the corresponding authors.

## Supplementary Materials

The following supporting information can be downloaded at: https://www.mdpi.com/article/10.3390/foods15071260/s1, Figure S1: Liver injury score. * *p* < 0.05, ** *p* < 0.01, and *** *p* < 0.001; Figure S2: Bar chart of LDA effect values for the indicator species. (A) LB group, (B) LC group.
