# Supplementary material for: Alcohol-Induced Acute Liver Disease in Mice: A Comparison of the Preventive Effects of Fermented Milk from Lactobacillus delbrueckii Subsp. bulgaricus or Lacticaseibacillus casei
Source: Foods. 2026 Apr 7;15(7):1260. doi: 10.3390/foods15071260 (PMC13073978; doi:10.3390/foods15071260)
Supplement: Supplementary file 1 [file foods-15-01260-s001.zip › foods-4209065-supplementary.pdf]

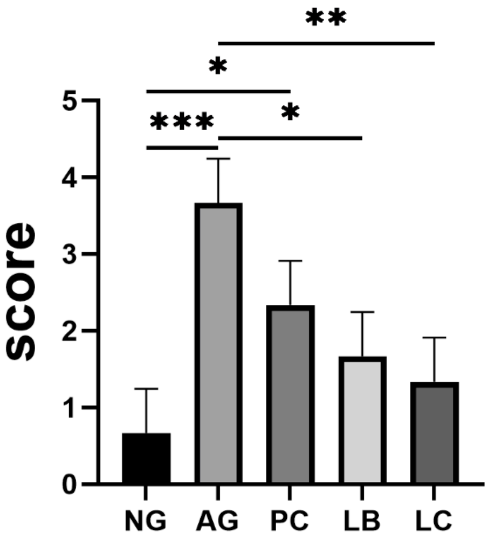

Figure S1 Liver injury score. *p < 0.05, **p < 0.01, and ***p < 0.001

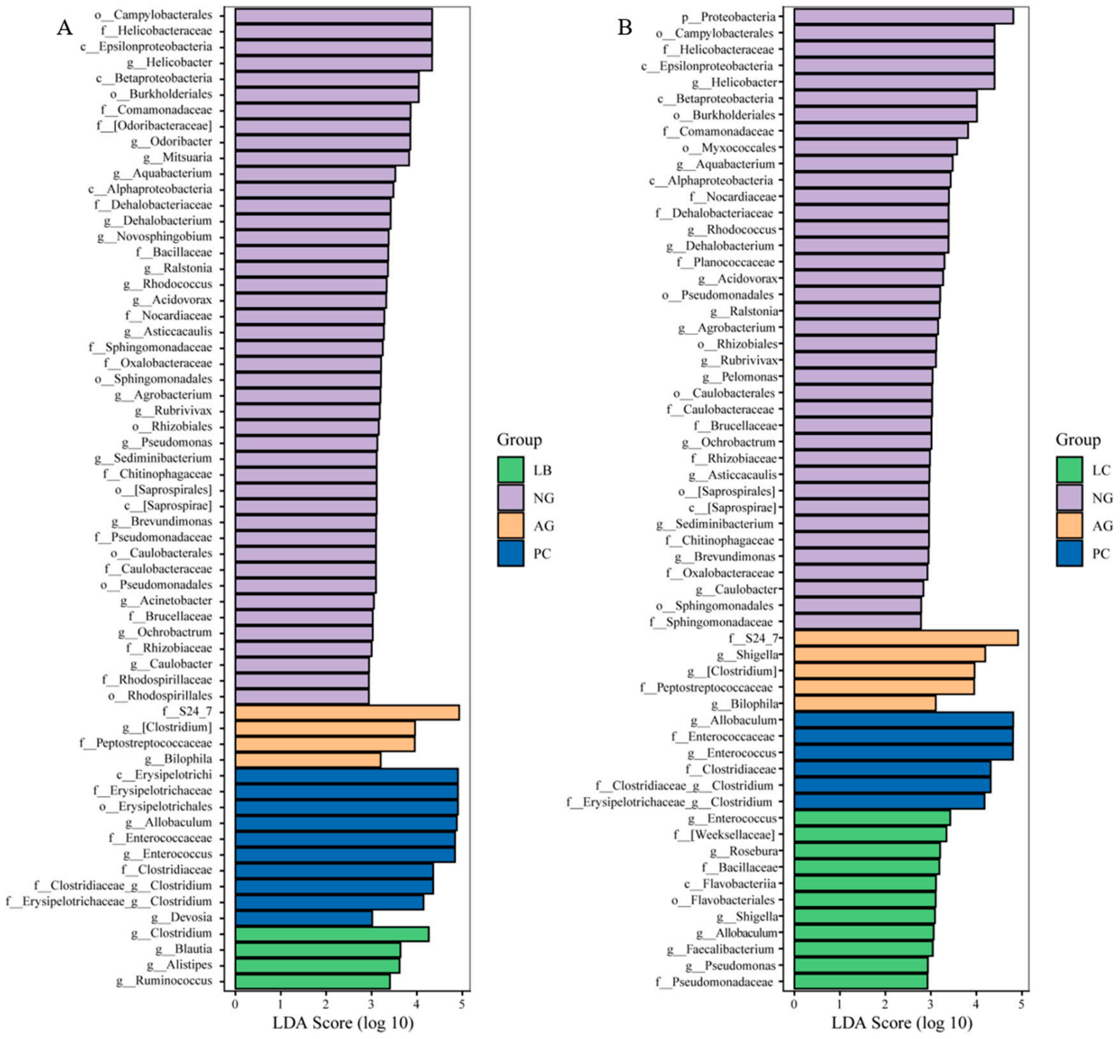

Figure S2 Bar chart of LDA effect values for the indicator species
